# Molecular circuit for exponentiation based on the domain coding strategy

**DOI:** 10.3389/fgene.2023.1331951

**Published:** 2024-01-23

**Authors:** Chun Huang, Xiaoqiang Duan, Yifei Guo, Panlong Li, Junwei Sun, Jiaying Shao, Yanfeng Wang

**Affiliations:** ^1^ School of Electrical and Information Engineering, Zhengzhou University of Light Industry, Zhengzhou, China; ^2^ Zhengzhou Kechuang Electronics Co., Ltd., Zhengzhou, China

**Keywords:** DNA strand displacement, domain coding, molecular circuit, mapping module, exponentiation

## Abstract

DNA strand displacement (DSD) is an efficient technology for constructing molecular circuits. However, system computing speed and the scale of logical gate circuits remain a huge challenge. In this paper, a new method of coding DNA domains is proposed to carry out logic computation. The structure of DNA strands is designed regularly, and the rules of domain coding are described. Based on this, multiple-input and one-output logic computing modules are built, which are the basic components forming digital circuits. If the module has n inputs, it can implement 2^*n*^ logic functions, which reduces the difficulty of designing and simplifies the structure of molecular logic circuits. In order to verify the superiority of this method for developing large-scale complex circuits, the square root and exponentiation molecular circuits are built. Under the same experimental conditions, compared with the dual-track circuits, the simulation results show that the molecular circuits designed based on the domain coding strategy have faster response time, simpler circuit structure, and better parallelism and scalability. The method of forming digital circuits based on domain coding provides a more effective way to realize intricate molecular control systems and promotes the development of DNA computing.

## 1 Introduction

In recent years, scientists have found that DNA, as the genetic material of life, has huge storage capacity, ultra-low power consumption, and fast computing speed, which makes it a good combination with computer science (Parker, 2003; Carell, 2011; Ramanamurthy et al., 2018; Zhao et al., 2021; Cao et al., 2023). Adleman (1994) successfully solved the directed Hamiltonian path problem using a DNA computing model in 7 days, which would take at least 2 years for the computer at that time. This shows that DNA has a great potential to deal with difficult mathematical problems (Chen et al., 2023). DNA strand displacement (DSD) is an efficient technology for DNA computing that provides rich intelligent toolboxes and has been widely used to solve many computational problems, such as NP problem (Lipton, 1995), information encryption (Sun et al., 2023; Wang et al., 2022a; Zou et al., 2022; Sun et al., 2023), nanomachines (Junke et al., 2020; Zhang et al., 2023b), intelligent drug delivery (Wang et al., 2022b), clinical diagnosis (Fan et al., 2022; Boskovic et al., 2023; Márquez-Costa et al., 2023), and biosensors (Dong et al., 2022; Xu et al., 2022; Sun et al., 2023). With the rapid development of DNA strand displacement technology, several molecular logic circuits have been designed (Wang et al., 2020; Zhu et al., 2020; Litovco et al., 2021; Chen et al., 2022). Seelig et al. (2006) designed AND, OR, and NOT gates with single nucleic acids as input and output signals and demonstrated signal recovery functions, which means arbitrary logic circuits can be constructed in theory. However, further study found that the NOT gate has a hidden danger of logic errors. The positive and negative values of the logic signal are distinguished by the level of concentrations, so the NOT gate can generate an “on” signal before receiving the output from the upstream gate, which leads to the wrong result. Qian and Winfree (2011) successfully solved the above problem by adopting the dual-track strategy. To a certain extent, it promotes the development of molecular logic circuits; a series of molecular circuits have been constructed, and the scale of circuits is becoming more extensive, for example, the five-bit cube root circuit (Wang et al., 2018), decoder circuit (Yang et al., 2016), four arithmetic circuits of addition, subtraction, multiplication, and division (Zou et al., 2017; Xiao et al., 2020), and timer circuit (Fern et al., 2017). However, with further research, we find that it is necessary to construct increasingly complex and large-scale practical logic circuits. It is known that the stability and accuracy of the dual-track circuit are obtained by increasing the complexity of the digital circuit. The number of gates in the dual-track circuit is twice that of the single-track digital logic circuit. The larger the scale of the circuit, the more complicated it is, and the more DNA strands are needed, which increases the difficulty of experiments and the system response time and even makes it difficult to realize. In the long run, the method based on a dual-track strategy limits the development of large-scale molecular logic circuits.

Aiming at the above issues, we adopt the domain coding strategy to construct DNA molecular circuits. Instead of using concentration to represent signal values like the dual-truck strategy, we define the logic value of the signal by coding the specific domains of the DNA single strand, which fundamentally solves the problem of NOT gate instability caused by concentration. Next, the multiple-input–single-output logic operation modules are designed based on the domain coding strategy, which are the basic components forming logic circuits. They are called mapping modules in this paper. A module can implement multiple logical functions. If the module has n inputs, it can perform 2^*n*^ logic functions, which reduces the difficulty of designing circuits and simplifies the structure of circuits. In addition to this, the mapping modules have good parallelism and encapsulation, strong expansibility, and remarkable programming characteristics, which show more advantages in constructing large-scale molecular logic circuits. In order to maintain stability and increase the speed of reaction among DNA strands, the fan-out gate and amplifying gate are also constructed using the domain coding strategy. Moreover, the report gate is designed to check the final result using the typical fluorescent labeling method. The domain coding strategy and the modularization design thought proposed in this paper provide a novel method to fabricate large-scale molecular circuits. At the same time, compared with the traditional dual-track strategy, the system response time is greatly shortened.

## 2 Domain coding rules and signal transmission

**FIGURE 1:**
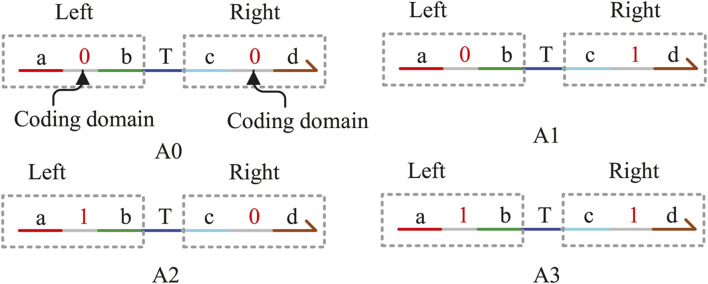
Signal strands with domain coding.

In this study, the DNA signal strand is expressed in the form of domain coding. As shown in Figure 1, the domain coding strand consists of four single strands, namely, A0, A1, A2, and A3. Each strand is divided into two parts by “T,” acting as the middle fulcrum. The left and right sides are composed of three base domains. The middle domain of the three base domains is the coding domain and can be coded as logic “0” or “1.” According to the coding principle of DNA strands, each strand includes two coded domains, such as the strands A0, A1, A2, and A3, which can be defined as (0,0), (0,1), (1,0), and (1,1), respectively. The left coded domain is used to hybridize with the upstream DNA strand, which includes the same domain, and the right-coded domain is used to determine the logical value of the strand. For example, if the coded value of the middle domain on the right is “0,” the DNA strand represents logic “0.” Conversely, the right domain “1” represents logic “1.” We can find that the logical value is not expressed by the concentration of the DNA strands any more but by coding “0” and “1” on the strands to determine the logical value, which can solve the difficult problem of NOT gate instability fundamentally.

## 3 Construction of auxiliary logic gates with domain coding

### 3.1 Fan-out gate with domain coding

**FIGURE 2:**
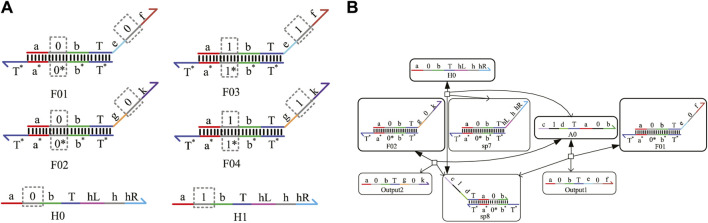
Fan-out-two gate. **(A)** Fan-out-two gate signal strands with domain coding; **(B)** reaction process of the fan-out-two gate with domain coding.

No matter whether in electronic circuits or DNA molecular circuits, one signal needs to fan out multiple signals representing the same logical value for different gates; meanwhile, zero interference between each module circuit must be guaranteed. The circuit responsible for realizing the function is called the fan-out gate in the DNA molecular circuit, which plays an important role in the whole logic circuit. In this study, the domain coding strategy is used to construct the fan-out gate. We take an instance of fanning out two signal strands to illustrate their composition and the whole reaction process. Figure 2A shows four double strands labeled F01, F02, F03, and F04, and two fuel strands, H0 and H1. Figure 2B shows the process of fanning out two logic 0 signal strands. It can be seen from the figure that after the input strand A0 representing logic 0 reacts with F01 and F02 separately, the Output1 and Output2 representing logic 0 are generated, which are the target strands and will be used to input downstream strands. The intermediate product sp8 is also generated at the same time, and then it reacts with the fuel strand H0 and outputs the input strand A0 again. This process shows that the input strand A0 reacts with the fan-out-two gate acting as a catalyst without being consumed and then receives two DNA single strands representing logic “0,” realizing the function of the fan-out-two gate. Herein, the concentration of the initial strands is set to 1X (1*X* = 10^4^ *nM*), and the concentration of the fuel strand H0 is twice that of F01 and F02.

### 3.2 Amplifier with domain coding

**FIGURE 3:**
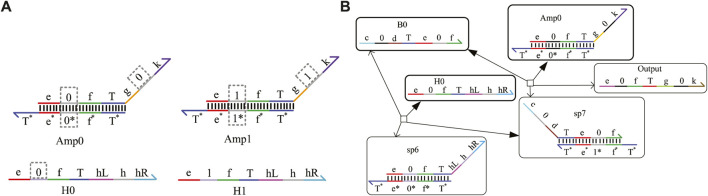
Amplifier with domain coding. **(A)** Domain coding strands of the amplifier; **(B)** reaction process of the amplifier with domain coding.

In the DNA chemical reaction network, the concentration of the DNA signal strand decays with the progress of the reaction, which affects the reaction rate of the whole system and delays the output time. It is necessary to add an amplifier in the appropriate position of the reaction network to transfer enough energy to the downstream module. A domain coding amplifier is shown in Figure 3A, which is mainly composed of two double strands, Amp0 and Amp1, and two fuel strands, H0 and H1. The combination of Amp0 and H0 completes the amplification task of logic “0,” and the combination of Amp1 and H1 completes the amplification task of logic “1.” Here, the input strand concentration is set as same as the amplifier double strand concentration, and the fuel strand concentration is set to twice the double-strand concentration. As shown in Figure 3B, the input strand B0 representing logic 0 from the upstream will react with double-strand Amp0, including the coded domain “0” and fuel strand H0, and generate the output strand representing logic “0,” while double-strand Amp1, including domain code “1” and fuel strand H1, does not participate in this reaction. The concentration of the strand B0 will be amplified to be consistent with the double-strand concentration. After the fuel strand reacts with the intermediate product strand sp7, the input strand B0 is generated again. So, the input strand B0 is used as a catalyst without being consumed. Adding an amplifier to the reaction may prolong the reaction time. Therefore, whether it is necessary to add an amplifier or at which position in the system depends on the specific situation.

### 3.3 Reporter gate with domain coding

**FIGURE 4:**
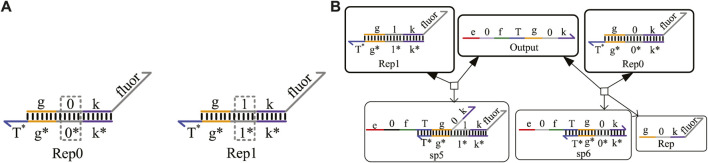
Reporter gate with domain coding. **(A)** Structure of the reporter gate strands; **(B)** reaction process of the reporter gate with domain coding.

To obtain the results of DNA logical computing by biochemical experiments, reporter gates constructed by fluorophore and quench agents are generally adopted for detection. As shown in Figure 4A, the reporter gate with domain coding is composed of two double-stranded strands, Rep0 and Rep1. The middle domain of the gray part is used to distinguish different logic signal strands. The “fluor” domain is composed of a fluorescence quenching group, which can convert the concentration of the output strand into a fluorescence signal of different colors. The process of obtaining results is shown in Figure 4B.

## 4 Designing of logic circuits based on the domain coding strategy

This paper proposes a novel method, domain coding strategy, to construct AND, OR, NOT, adder, subtractor, and other complex logic circuits. In order to simplify the circuit structure and realize the generality of circuit modules, multiple-input and one-output modules will be built using mathematical mapping mechanisms, which are the basic elements forming DNA logic circuits. We can define a function from A to A, where f(a) = f(b) = A. If set A = a, b, f(a) can match map a or b. In the same way, f(b) can also map a or b. From the above derivation, we can draw the conclusion that there are 2^2^ mapping situations for set A with two elements. If set *A* ^*n*^ = *A* × *A* × *A* … × *A*, the set *A* ^*n*^ contains |*A*|^*n*^ elements according to the Cartesian product; each element may correspond to any member of the set A, and each element of the set *A* ^*n*^ has |*A*|^*n*^ possible correspondence, so the count of mappings from set *A* ^*n*^ to set A is $|A{|}^{|A{|}^{n}}$ . If A = [a,b], n = 1, the count of mappings is $2^{2^{1}}=4$ , which is defined as a one-input mapping module. If A = [a,b], n = 2, the count of mappings is $2^{2^{2}=16}$ , which is defined as a two-input mapping module. If A = [a,b], n = m, the count of mappings is $2^{2^{m}}$ , which is defined as an m-input mapping module. Logic function modules can be realized based on the mapping relationships of the m-input mapping module.

### 4.1 One-input mapping module

**FIGURE 5:**
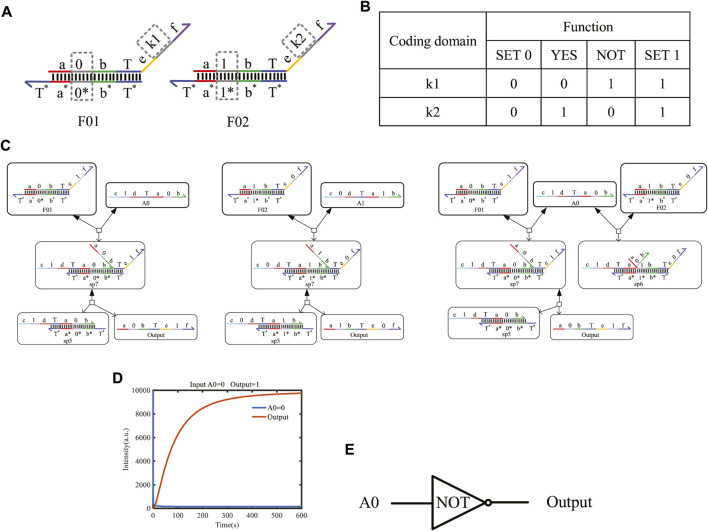
One-input mapping module. **(A)** Structure of one-input mapping module. **(B)** Specific functions implemented by the one-input mapping module. **(C)** The input strand reacts with the double strands of the one-input mapping module to produce the output strand of logic “1” and logic “0.” **(D)** Simulation result of the NOT gate. **(E)** Symbol of the NOT gate.

The one-input mapping module includes two double strands, F01 and F02, as shown in Figure 5A. The double-strand F01 only receives the input signal strand representing logic “0,” and the strand F02 only reacts with the input signal strand representing logic “1.” The domains “k1” and “k2” can be coded logic “0” or logic “1” and determine the logical value of the output strands. Based on the above mapping method, the domains k1 and k2 can be mapped to four situations and implement four logic functions. See Figure 5B for specific functions. When k1 = 0 and k2 = 0, the one-input mapping module can output logic “0” regardless of whether the input signal is logic “0” or logic “1.” Here, the function is called “SET 0.” When k1 = 1 and k2 = 1, the one-input mapping module can output logic “1,” regardless of whether the input signal is logic “0” or “1.” Here, the function is called “SET 1.” When k1 = 0 and k2 = 1, the one-input mapping module outputs the same logic value as the input signal. Here, the function is called “YES.” When k1 = 1 and k2 = 0, the one-input mapping module outputs the opposite logic value to the input signal. Here, the function is called “NOT.”

Figure 5C shows the chemical reaction of a one-input module. Taking k1 on F01 as “1” and k2 on F02 as “0,” the logical value of A0 is flipped to the logic “1,” and the logical value of A1 is flipped to the logic “0” after base-pairing with the input strand A0 representing logic “0” and A1 representing logic “1,” which realizes the logic inverse operation. Similarly, when the domain coding input strands react in parallel with the two DNA double strands, the toehold T exposed in the middle of the input strand will hybridize with the left coding domain of the two double strands. As shown in Figure 5C, the right coding domain of the input strand A0 is “0,” which is consistent with the left coding domain of double-strand F01. After hybridizing, a single strand will be obtained. Meanwhile, the coding domain on the left side of the double-strand F02 is “1,” which is inconsistent with the coding of the input strand A0; there is no single-strand output after the reaction, and only one waste sp6 is output.

In order to detect the output results more conveniently, an amplifier gate and a fluorescence report gate are added to amplify the output concentration and convert the concentration into the fluorescence intensity. Under the action of one input strand, the output fluorescence curve after a parallel reaction with two double strands is shown in Figure 5D. The initial concentration of the reactants is set to 1X nM (1X = 104 nM), and we find that the output shows high-intensity fluorescence and reaches a stable state after 400 s. This simulation confirms that the two double strands of the one-input mapping module participated in the reaction in parallel, which improves the reaction speed and has good parallelism.

### 4.2 Two-input mapping module with domain coding

**FIGURE 6:**
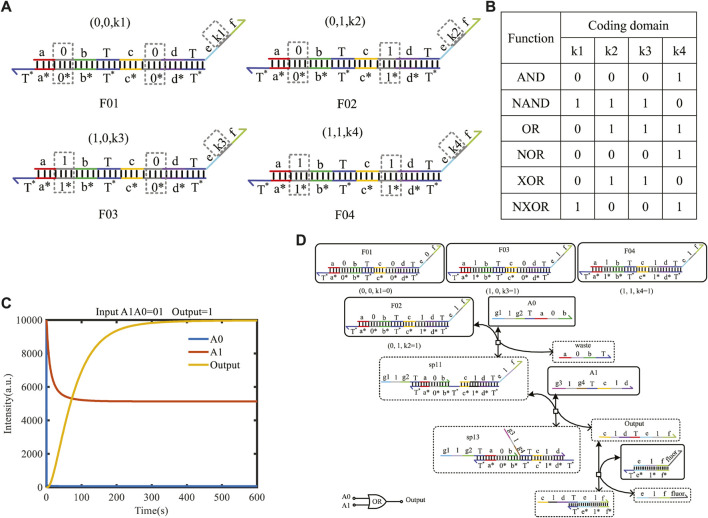
Two-input mapping module. **(A)** Double-strand structure of the two-input mapping module; **(B)** part of functions implemented by the two-input mapping module; **(C)** simulation result of the OR gate when the logic value of input strands A1A0 is 01, and the logical symbol of the OR gate; **(D)** reaction process of the OR gate implemented by the two-input mapping module.

The two-input mapping module is composed of four double strands; each strand includes one bare toehold “T” and three coding domains, as shown in Figure 6A with dotted boxes. The coding domains of the left domains can be coded as (0, 0), (0, 1), (1, 0), and (1, 1), and similarly, the coding domains k1, k2, k3, and k4 of the right domain can have 16 cases. Combined with the left domains and coding the domains k1, k2, k3, and k4 on the mapping module, the 16 different logical functions can be implemented. Some of the classic functions are shown in Figure 6B.

Here, taking A0 as logic “0” and A1 as logic “1,” the initial species concentration is defined as 1X nM (1*X* = 10^4^ nM). From Figure 6C, the input strand A0 only reacts with F02 belonging to the two-input mapping module and generates the intermediate reactant. Then, the strand sp11 reacts with the input strand A1 for secondary base-pairing, and the output of logic “1” is obtained. This process verifies the realization of the logic function of the OR gate. To facilitate the detection of output results, the amplifier gate and the report gate are added here. A fluorescence curve is shown in Figure 6D, which reached the dynamic balance after 300 s.

### 4.3 Three-input mapping module with domain coding

**FIGURE 7:**
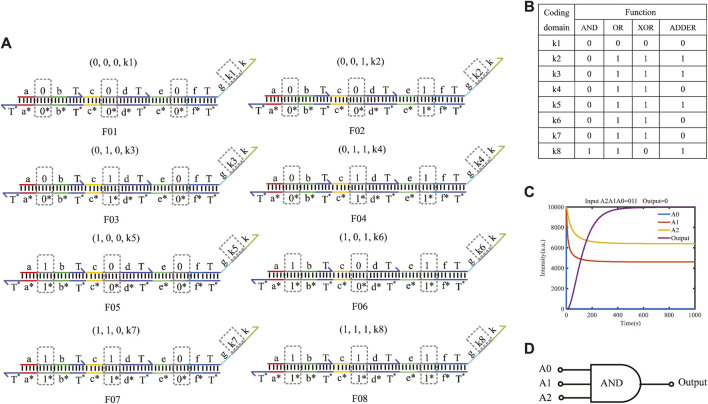
Three-input mapping module. **(A)** Double-strand structure of the three-input mapping module; **(B)** part of functions of three-input module; **(C)** simulation result of the AND gate built by the three-input mapping module; **(D)** logical symbol of the AND gate.

The three-input mapping module is composed of eight domain-coding double strands and can implement 2^8^ different logic functions. Figure 7A shows the structure of three-input mapping modules. Different logic functions can be realized by taking various values of the coding domains k1–k8 on the double strands. Figure 7B shows some of the functions implemented by the three-input module.

Here, taking the input logic value “011” as an example, the concentration of double strands is defined as 1X nM (1*X* = 10^4^ nM). By coding the value of k1–k8 on the double strands as (0, 0, 0, 0, 0, 0, 0, 1), the AND gate function of three inputs and one output can be implemented. The simulation result is shown in Figure 7C. The results show that when the logical value of the input strands A2A1A0 is 011, eight double strands participate in the reaction in parallel in the whole reaction process, and only one double strand reacts with the input strand to get the output result of logic “0,” which verifies the logical function of the AND gate. The fluorescence intensity of the outputs enters dynamic equilibrium after 400 s. As long as the molecular circuit conforms to the feature of three inputs and one output, the logic functions can be implemented by a three-input mapping module. Similarly, if the molecular circuit needs n input signals, we can get 2^*n*^ types of double strands to easily form an n-input mapping module, which can realize $2^{2^{n}}$ types of logic functions.

### 4.4 Construction of molecular circuits for square roots

**FIGURE 8:**
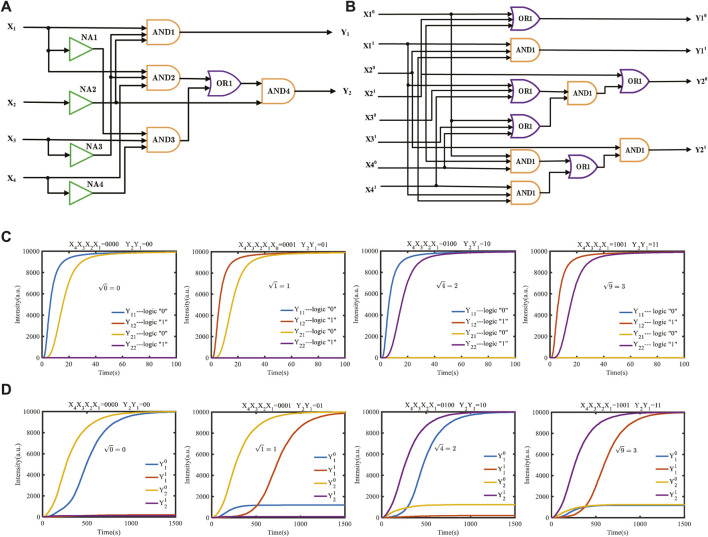
Molecular logical circuit for the calculation of square roots. **(A)** Domain-coding logical circuit for calculating the square root; **(B)** dual-track logic circuit to carry out the equal square-rooting function; **(C)** simulation results with four typical inputs using the domain coding strategy; **(D)** simulation results with four inputs using the dual-track strategy.

To further demonstrate the power of the domain coding strategy to perform complex operations, we construct a square root circuit for the four-bit binary input, as shown in Figure 8A. The typical input values 0, 1, 4, and 9 are calculated, and the simulation result is shown in Figure 8C. Figure 8B shows a square-root circuit constructed using the dual-track strategy. The simulation conditions are completely equal between them. The initial concentration of reaction species is set to 10,000 nM, and the binding and unbinding rates are 3.0e-4 *nM* ^−^1*s* ^−^1 and 0.1126 *s* ^−^1, respectively. The output curves consist of four types of fluorescence curves: *Y*_1_1, *Y*_1_2, *Y*_2_1, and *Y*_2_2, which are dyed to different colors. The output curves *Y*_1_1 and *Y*_2_1 represent logic “0,” and *Y*_1_2 and *Y*_2_2 represent logic “1.” For example, the graph shown in Figure 8C includes a blue curve “*Y*_1_1” and a yellow curve “*Y*_2_1,” which means that the output “*Y*_1_ *Y*_2_” is “00” in binary; that is, when the input decimal number is “0,” the square root result is “0.”

The simulation results of the comparison of the domain-coding square-root circuit with the dual-track logic circuit under the equal simulation condition demonstrated that the dual-track logic circuit requires approximately 130 signal strands to perform a calculation, while the domain-coding logic circuit only needs 64 signal strands to perform the identical function, which decreases the number of strands by nearly 50%. As shown in Figures 8C, D, the calculation time of a dual-track logic operation is 1,500 s, while the domain-coding circuit only needs 60 s, which is nearly 30 times higher than the dual-track circuit.

### 4.5 Construction of a molecular logical circuit for exponentiation

Exponentiation is important for computing; many intricate practical problems can be solved by exponentiation, such as energy calculation problems, the calculation times of supercomputers, and modular exponentiation in the core algorithm of RSA. In this study, we adopt the input mapping modules using the domain coding strategy to construct an exponentiation circuit that can achieve a power operation of 2^0^ 2^7^. The simulation results show that the DNA molecular circuit designed using the domain coding strategy has the advantages of faster operation speed, simpler circuits, and better parallelism than a dual-track molecular circuit, which can be used as a reference for the subsequent construction of complicated molecular circuits.

**FIGURE 9:**
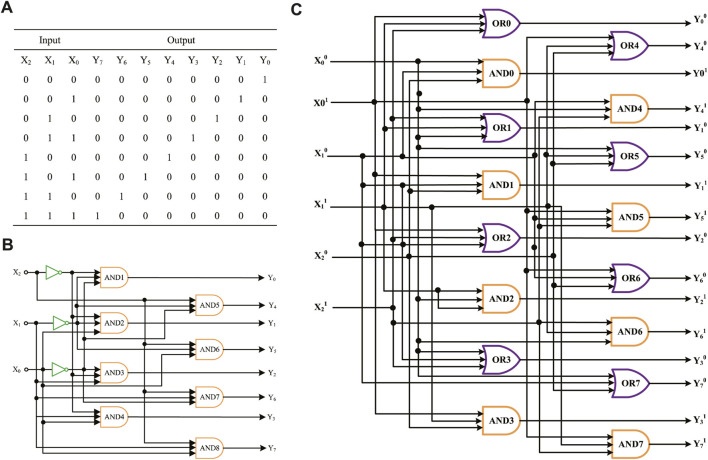
Molecular logical circuit of exponentiation. **(A)** Truth table of exponentiation; **(B)** logical circuit of exponentiation designed using the domain coding strategy; **(C)** logic circuit of exponentiation designed using the double-track strategy.

According to the exponentiation algorithm, the truth table of power operations can be ascertained, as shown in Figure 9A. Suppose that the input signal is *X*_2_ *X*_1_ *X*_0_, which means eight different exponents of 2, and the output signals are *Y*_7_ *Y*_6_ *Y*_5_ *Y*_4_ *Y*_3_ *Y*_2_ *Y*_1_ *Y*_0_, representing the exponentiation result. For example, if you enter *X*_2_ *X*_1_ *X*_0_ as 010 in binary, which means the input is 2^2^ in decimal, the result *Y*_7_ *Y*_6_ *Y*_5_ *Y*_4_ *Y*_3_ *Y*_2_ *Y*_1_ *Y*_0_ is 00001000 in binary, which means the output result is 4 in decimal. As shown in Figure 9B, the logic circuit of exponentiation contains three NOT gates and eight AND gates. We use three one-input mapping NOT gate modules and eight three-input mapping AND gate modules to accomplish the operation.

Figure 9C shows an exponentiation logic circuit constructed using the dual-track strategy; it uses eight AND gate modules and eight OR gate modules. In terms of the number of logical gates, the molecular logic circuit using the domain coding strategy has fewer logical gates than the dual-track circuit. To sum up, the domain coding strategy can reduce circuit complexity, save on economic costs, and have potential for expanding complex molecular logic circuits.

**FIGURE 10:**
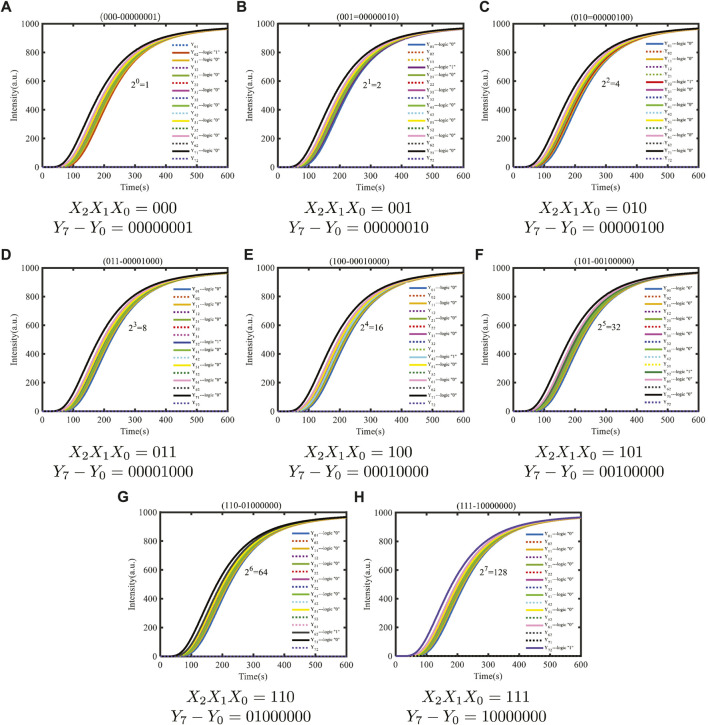
Dynamic simulation of exponentiation based on the domain coding strategy.

The DSD simulation result is shown in Figure 10. The initial concentration of the input signal strands and double strands included in the input mapping module is set to 1,000 nM, and the concentration of the fuel strand for the fan-out gate is set to twice the sum of the fan-out gate double strands. The concentration of the fuel strand for the amplifier is set to be twice that of amplifier double strands. As shown in Figure 10, *X*_2_ *X*_1_ *X*_0_ represent the input strands of domain coding, and eight different logic cases can be taken. The logical values of the output terminal *Y*_7_ − *Y*_0_ are represented by the two fluorescent signal strands *Y*_*X*1_ and *Y*_*X*2_ (X = 0, 1,.7), respectively. The fluorescent signal strand *Y*_*X*1_ represents the logical value “0,” and *Y*_*X*2_ represents the logical value “1.” As shown in Figure 10A, the logical value of the three input strands, *X*_2_ *X*_1_ *X*_0_, is 000, which means that the corresponding output strands *Y*_7_ − *Y*_0_ should be 00000001 when the E-operation is taken as 2^0^ = 1. Therefore, the output strands *Y*_02_, *Y*_11_, *Y*_21_, *Y*_31_, *Y*_41_, *Y*_51_, *Y*_61_, and *Y*_71_ will output high-intensity fluorescence curves, and the output strands *Y*_01_, *Y*_12_, *Y*_22_, *Y*_32_, *Y*_42_, *Y*_52_, *Y*_62_, and *Y*_72_ will output low-intensity fluorescence curves equal to the horizontal axis. Owing to the fact that the concentration of the DNA strands is gradually consumed during the biochemical reaction, eight domain-coding amplifiers are joined to amplify the concentration of output strands, and eight fluorescence reporter gates are joined to finish the detection of the outputs.

According to the simulation results, under the default binding and unbinding rates of DSD software, the simulation results are consistent with the theoretical results, proving that the E molecular circuit constructed using the domain coding strategy is feasible. The whole time of E molecular circuits to complete E operation is only 600 s, and the number of initial species involved in the calculation is 71. Compared with the previous molecular circuits, the reaction time is greatly shortened, and the circuit structure is simplified, which is more conducive to the development of modular molecular circuits.

## 5 Conclusion

In this paper, a general strategy, the domain coding strategy, is presented, which is utilized to design molecule logic circuits for implementing arbitrary digital DNA computing. The structure design of DNA strands based on the domain coding strategy is regular, and the position of coding domains is fixed, which makes the DNA strand design simple. The logical value of a single DNA strand is defined according to the right domain code instead of the level of DNA strand concentration. If the domain code is “1,” the strand represents the logic value “0.” If the domain code is “2,” the strand represents the logic value “1.” Without increasing the number of circuit gates, the method eliminates the problem of the NOT gate output error caused by using high and low concentrations to represent the logic value in the previous molecular circuits. In order to speed up the biochemical reaction and stabilize the desired circuits, a fan-out gate and an amplifying gate using the coding domain strategy are constructed, which can be added to any position of the DNA circuits, depending on the demand. In addition, the modularity can be reflected in the multiple-input mapping module using the domain coding strategy. One mapping module can carry out multiple logic computing. If the mapping module has n inputs, it can realize $2^{2^{n}}$ types of logic functions, which optimizes the structure of digital circuits and offers an efficient way for large-scale and achievable molecular circuits.

In order to further verify the superiority of the domain coding strategy, a four-bit square-root circuit and an exponentiation circuit are constructed. Compared with the dual-track strategy, many advantages are obtained, such as the improvement in running speed. Using the domain coding strategy, the stabilizing time is often several hundreds of seconds, while it can reach 4,000 s or more using the dual-track strategy under the same experimental conditions for the identical logic function. Moreover, the needful components are reduced by nearly half compared with the dual-track strategy, which can reduce the amount of material and optimize the design. In conclusion, the domain coding strategy, with its strong flexibility and programmability, promotes the development of biological analysis technology, which has great potential in the construction of complex cascade molecular circuits and is expected to contribute to the realization of molecular systems with comprehensive decision-making functions.

## Data Availability

The original contributions presented in the study are included in the article/Supplementary Material; further inquiries can be directed to the corresponding author.
